# Serum Albumin in Nasal Drug Delivery Systems: Exploring the Role and Application

**DOI:** 10.3390/pharmaceutics16101322

**Published:** 2024-10-11

**Authors:** Sandra Aulia Mardikasari, Gábor Katona, Ildikó Csóka

**Affiliations:** 1 Institute of Pharmaceutical Technology and Regulatory Affairs, Faculty of Pharmacy, University of Szeged, Eötvös St. 6, H-6720 Szeged, Hungary; sandraaulia@unhas.ac.id (S.A.M.); csoka.ildiko@szte.hu (I.C.); 2 Faculty of Pharmacy, Hasanuddin University, Makassar 90245, Indonesia

**Keywords:** albumin, nasal delivery, albumin nanoparticles, mucin, nasal formulation

## Abstract

The application of serum albumin in various types of formulations has emerged as a valuable option in biomedical research, especially in the field of nasal drug delivery systems. A serum albumin-based carrier system has been employed due to several benefits, such as enhancing drug solubility and stability, generating the desired controlled release profile, and developing favorable properties with respect to the challenges in nasal conditions, which, in this case, involves hindering rapid elimination due to nasal mucociliary clearance. Accordingly, considering the important role of serum albumin, in-depth knowledge related to its utilization in preparing nasal drug formulation is highly encouraged. This review aimed to explore the potential application of serum albumin in fabricating nasal drug formulations and its crucial role and functionality regarding the binding interaction with nasal mucin, which significantly determines the successful administration of nasal drug formulations.

## 1. Introduction

Serum albumin, a predominant and essential protein within the human body, fulfills a pivotal function in sustaining intravascular colloid osmotic pressure, detoxifying harmful substances, and conveying therapeutic compounds. Owing to its inherent biocompatibility and biodegradability, albumin is extensively utilized in various medical applications [1]. The protein’s substantial affinity for binding both lipophilic and hydrophilic pharmaceuticals, coupled with its low toxicity and immunogenicity, has gained significant interest for drug delivery purposes [2,3,4]. In light of these attributes, the deployment of albumin-based nanoparticulate systems as drug carriers has been increasingly recognized due to their advantageous properties, such as high drug encapsulation efficiency, the capability to augment drug solubility and stability, and the provisions of a regulated drug release profile [5,6]. Accordingly, there has been much research investigating the advantages of albumin-based nanoparticles for different therapeutic applications, including for nasal drug delivery [7,8,9,10].

Nasal drug delivery refers to the administration of drugs through the nasal cavity. It offers several advantages, including non-invasiveness, rapid onset of action, and targeted delivery [11,12]. Nasal drug delivery can be employed for the purpose of both local effects (such as treating allergic rhinitis or nasal congestion) and systemic effects (such as cardiovascular indications) [13,14]. Additionally, it has gained interest in terms of delivering drugs to the central nervous system (CNS) through nasal pathways, especially in the field of nose-to-brain delivery [15,16,17,18]. Besides many advantages, nasal route delivery also faces many challenges, such as mucociliary clearance, specific nasal pH, nasal temperature, ionic and enzymatic conditions, etc. [19]. Therefore, the strategy of formulation to overcome those obstacles is of great importance for ensuring therapy effectiveness.

In this circumstance, several pieces of nasal delivery research have employed serum albumin as a delivery carrier in the form of albumin-based nanoparticles [20] that encapsulate the active substance [21]. Remarkably, this approach has shown promising results regarding its potential to overcome nasal route delivery challenges, especially its properties in avoiding rapid elimination from the nasal cavity, thus enhancing the residence time of the formulation on the nasal mucosa [2,6,9]. However, the significant role of serum albumin, along with its advantages and properties as a suitable carrier for nasal drug delivery, have not been widely discussed.

Therefore, this review aims to explore the valuable role of serum albumin as a suitable nasal carrier system and also its various applications in nasal drug formulation. At first, an overview of serum albumin properties and binding capabilities is discussed. Subsequently, the advantages and challenges of nasal drug delivery systems are also presented. Afterward, the role of serum albumin in nasal delivery systems, as well as various applications of albumin-based nanoparticles for nasal drug delivery, are pointed out. Finally, the methods for fabricating albumin-based nanoparticles are explained. To our knowledge, this work is the first review that highlights the significant role and potential of serum albumin for application in nasal drug delivery systems. Substantially, a proper understanding with respect to the characteristics of serum albumin as a potential carrier system might help in overcoming nasal drug delivery challenges and in developing appropriate nasal drug formulation in order to obtain the desired therapeutic results.

## 2. Overview of Serum Albumin

### 2.1. Structural Characteristics

**Figure 1:**
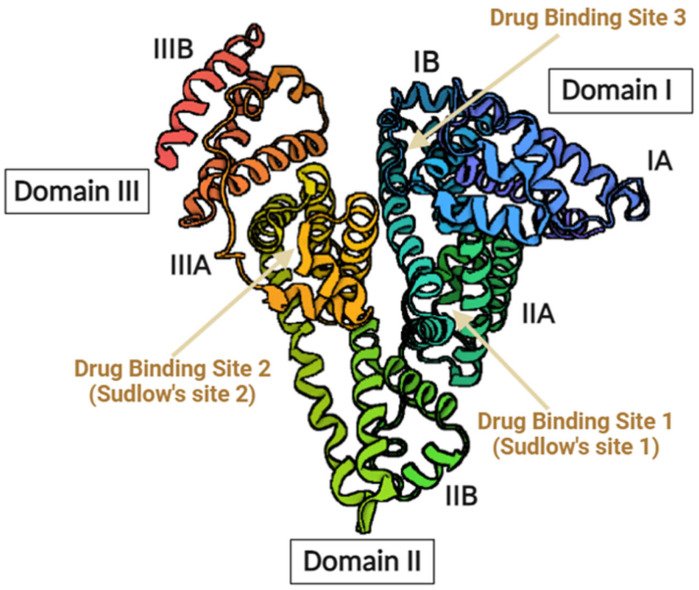
Albumin structure with three specific domains and drug-binding sites [25,26,27,28]. This figure was created through Biorender.com.

Albumin makes up about 60% of all plasma proteins and is the most prevalent protein in blood plasma. With a molecular weight of 67 kDa and an average half-life of 19 days, it is a small globular protein that is highly water-soluble [22]. The albumin structure has around three specific domains and binding sites, as shown in Figure 1. It exhibits stability within the pH range of 4–9 and can withstand up to 10 h of heating at 60 °C [22]. In addition to nourishing tissues and delivering hormones, vitamins, medications, and divalent cations like calcium and zinc throughout the body, albumin is also in charge of maintaining blood osmotic pressure. In inflammatory conditions, it functions as a free radical scavenger and is connected to wound healing and coagulation. There are several varieties of albumin, such as egg white-ovalbumin, mouse serum albumin, rat serum albumin, rabbit serum albumin, human serum albumin (HSA), and bovine serum albumin (BSA). However, HSA and BSA are the most widely used serum albumin types in the drug delivery research field [23].

HSA is a globular protein in blood plasma with a well-defined three-dimensional shape that contains around 585 amino acids, with levels ranging from 3.5 to 5 g/dL. HSA is produced by hepatocytes in the liver at a rate of 9–12 g/day. Its half-life in the biological system is 19 days, but, in circulation, it lasts only 16–18 h [22]. HSA assembles into a heart-shaped molecule composed of three homologous domains with a 67% α helix and disulfide bridges [24]. Each domain consists of two subdomains that share common structural motifs. Moreover, HSA contains several long α-helices, which contribute to its relatively static shape [25]. This structural stability is essential for regulating blood pressure and maintaining its functional properties. Regarding binding domains, HSA has eleven distinct binding domains for hydrophobic compounds. These domains allow HSA to interact with various ligands, including fatty acids, hormones, drugs, and metal ions. Interestingly, HSA can simultaneously bind multiple ligands. For example, one hemin molecule and up to six long-chain fatty acids can bind to HSA concurrently. Overall, the structural features of HSA enable it to perform critical functions, including ligand transport, regulation of blood pressure, and scavenging of reactive oxygen species [24].

Furthermore, another type of serum albumin, which is specifically derived from bovine, namely, BSA, shares many similarities with the characteristics of HSA, including a molecular weight of approximately 69.323 kDa and an isoelectric point (pI) of 4.7 in water at 25 °C [22,24]. This means that the protein is negatively charged at a neutral pH and positively charged in acidic conditions. The binding of both positively and negatively charged substances can occur from the presence of both positively and negatively charged amino acids in BSA [29]. Moreover, BSA has been widely used as a drug delivery carrier due to its low cost, wide availability, and ease of purification. Its high loading capacity, water solubility, and ability to bind both hydrophilic and hydrophobic drugs make it an extremely versatile carrier [30,31,32]. However, the sole drawback might be the potential for an immunogenic response in vivo in mice as well as humans. There is an 80% sequence homology between BSA and HSA, a less than 1% difference in molecular weight, and the same isoelectric points. The quantity of tryptophan (Trp) residues is one significant variation, in which the BSA has two Trp, while HSA possesses only one. Albumin has been used in many drug delivery systems, including cancer-targeted drug delivery systems [33,34,35].

### 2.2. Binding Properties and Utilization

Serum albumin is a multifaceted protein with a wide array of binding capabilities and a crucial role in the transport mechanism within the human body. Serum albumin can bind to a variety of endogenous and exogenous ligands, including fatty acids, hormones, drugs, and metal ions. Moreover, the binding of drugs to albumin is essential for understanding their pharmacokinetic profiles and influences their efficacy in vivo. Albumin’s drug-binding properties can affect the active concentration of drugs, impacting their therapeutic effectiveness [36,37]. Furthermore, as the main transport and reservoir protein in the circulatory system, albumin facilitates the movement of numerous substances throughout the body. Albumin interacts with cellular receptors that not only promote the uptake of drugs into cells but also contribute to its long circulatory half-life. This interaction is pivotal for the delivery of therapeutic agents to their target sites. Also, in terms of antioxidant properties, serum albumin can bind to reactive oxygen species, which are harmful to cell survival, thus playing a role in protecting cells from oxidative stress [38].

Moreover, the widespread distribution of albumin has been observed in the blood as well as in different interstitial fluids and organs. The body’s albumin distribution properties are expertly regulated by various albumin receptors. A variety of proteins, including FcRn, gp60 (Albondin), gp18, SPARC, cubilin, megalin, calreticulin, and CD36, are known as albumin receptors [39,40]. They have distinct in vivo tissue distributions with various albumin recognition sites. Thus, the distribution of albumin in vivo is cleverly regulated by these numerous albumin receptors. Cellular receptors that bind albumin are essential for the homeostatic control of albumin [41]. Examining these albumin receptors further establishes new opportunities for using albumin as a drug delivery vehicle [40,41].

The surface of endothelial cells in peripheral capillaries contains albumin transport pathways, including those mediated by gp60. The BBB stops albumin from crossing in vivo and in vitro, but brain endothelial cells express themselves relatively little. Recent studies have shown evidence of albumin uptake across the nasal epithelium, allowing it to avoid the BBB. Elevated levels were found in the olfactory bulb and striatum, with rapid uptake through a saturable transport system to all regions of the brain [42,43]. In summary, serum albumin’s binding capabilities and transport mechanism are integral to maintaining homeostasis and effective drug delivery within the human body. Its ability to interact with a wide range of substances makes it an indispensable protein in clinical applications [3].

Furthermore, serum albumin has a wide range of applications in biomedical research due to its unique properties and functions, as mentioned in Table 1. In the field of drug delivery systems, serum albumin is utilized as a carrier for various drugs, enhancing their solubility, stability, and bioavailability [6,44]. It can also facilitate targeted delivery to specific tissues or organs [34]. Further, serum albumin also contributes to tissue engineering for the development of scaffolds that support cell growth and tissue regeneration [45,46]. Serum albumin has been employed in cell culturing, storage, in vitro fertilization, and transplantation. Additionally, due to its natural presence in the human body, albumin is used in bioimaging techniques to improve the visualization of structures and functions within the body [20,47].

Serum albumin was also utilized for nanoparticle formation in the form of drug-containing albumin-based nanoparticles, which are engineered for diagnostic and therapeutic purposes, including cancer treatment, due to their biocompatibility and ability to carry therapeutic agents [34,48]. In line with this, albumin also possesses potential for immunological research, in which albumin’s role in the immune response is studied for its potential in vaccine development and understanding disease mechanisms. Lastly, as a reflection of various physiological states, albumin is used as a biomarker in the diagnosis and monitoring of diseases [1,20]. These applications demonstrate the versatility of serum albumin in advancing medical science and improving patient care.

It is worth mentioning that, given the obvious potential of serum albumin as a carrier system, there have been several clinically approved albumin-based formulations, such as for the treatment of cancer, hepatitis, and diabetes mellitus [5]. However, the clinical application of an albumin-based formulation that is specifically for nasal route administration is still unexplored. Therefore, extensive research with respect to the application of serum albumin for nasal drug delivery is highly encouraged to provide knowledge that can be useful for drug development in the future.

**Table 1:** Several applications of serum albumin in biomedical research field.

Sr. No	Research Field	Role	Ref.
1	Tissue engineering and cell transplantation	Albumin hydrogel	[49]
2	Carrier in nanodrug delivery	Albumin-based nanoparticles	[22]
3	Cancer therapy	Albumin-based nanoparticles	[34]
4	Antitumor activity	Albumin-based nanoparticles	[50]
5	Antitumor and tissue engineering	Albumin-based nanoparticles	[37]
6	Carriers for various delivery routes	Albumin-based nanoparticles	[3]
7	Cancer diagnosis and treatment	Albumin-based nanoparticles	[51]

Moreover, in terms of the utilization of serum albumin for nasal drug delivery, great potential results have been previously shown by several research groups. In 2010, research conducted by Dhuria et al. found that olfactory nerves could be a pathway for reaching the brain due to the high turnover of olfactory neurons and the presence of perineural channels formed by olfactory ensheating cells [52]. Also, in 2014, Falcone et al. demonstrated that albumin possesses the ability to reach the brain after intranasal administration, with the highest levels found in the olfactory bulbs [43]. Accordingly, these findings present the valuable capabilities of albumin and suggest that CNS-targeted drug delivery might be possible through nasal route application by utilizing serum albumin as a carrier system to deliver the drug for the treatment of neurological diseases [53,54].

The prospect of serum albumin as a promising carrier system for nasal drug delivery is highly appealing due to its wide application in the field of biomedical research and drug delivery alongside its binding affinity and valuable characteristics. This potential is further supported by its ability to access the brain region via the olfactory nerves, making it possible to achieve optimal therapeutic efficacy through nasal route administration.

## 3. Nasal Drug Delivery Systems

### 3.1. Advantages of Nasal Drug Delivery

**Figure 2:**
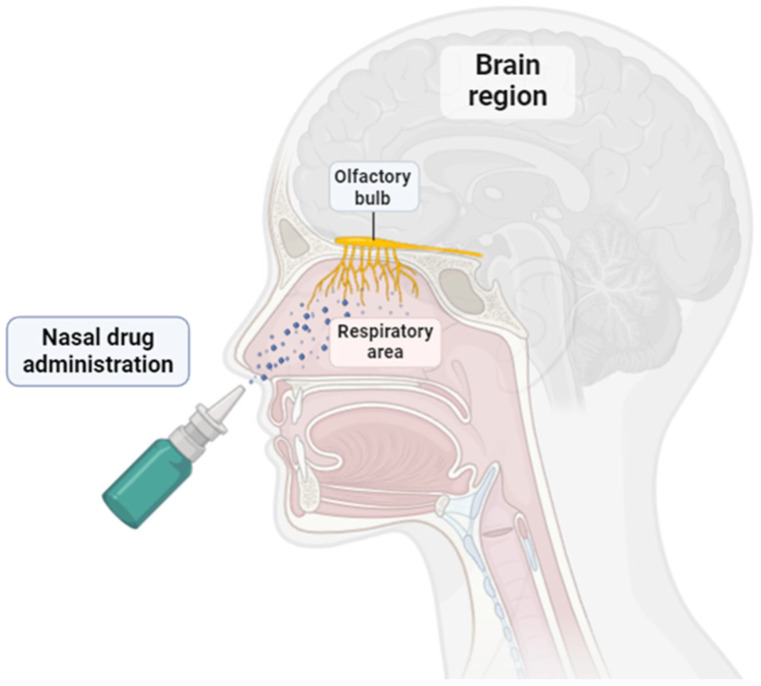
Schematic illustration of drug administration through nasal route pathway.

Nasal route delivery has emerged as an attractive delivery route for administration since many advantages are provided. As shown in Figure 2, the schematic illustration of the nasal drug delivery pathway involves the respiratory and olfactory area, in which the possibility of reaching the brain region is higher [55,56,57,58].

Nasal administration offers several advantages over other delivery methods, including the prevention of first-pass metabolism, the high permeability of some drugs in the nasal epithelium, rapid drug absorption through this membrane, rapid onset of action, improved patient compliance and comfort, and a sustained and prolonged effect [16,59,60]. The nasal mucosa has a strong blood supply, facilitating drug absorption and, consequently, a fast local and rapid systemic effect as well as providing high systemic blood levels [19,61,62].

Nasal drug absorption is primarily influenced by the physicochemical characteristics of the drugs [63]. In this circumstance, if the drug molecules are too large (more than 1 kDa), very lipophilic, or highly ionized, then the drug cannot pass through the nasal mucosa, and a large proportion could be pre-systemically removed or degraded [12,64]. Numerous factors can influence the delivery of drugs through the nasal passage. Drug stability and ionization level can be influenced by pH, which also might irritate the nasal mucosa. Formulations with excessively high levels of hyper- or hypotonicity can affect the ciliary movement, leading to reduced drug absorption [65,66,67,68]. Viscous formulations may be less absorbed because of the lower drug release, but they can be more easily retained on the nasal mucosa than liquid formulations [9,63]. The drug absorption process is also influenced by the surface, drug concentration and quantity, dosage form physical state, and head position during administration [64].

Nasal drug administration is non-invasive, painless, and easy to perform. It also carries a minimal risk of damage or infection from blood-borne illnesses like HIV or hepatitis B. It is possible to view the urgency of nasal delivery systems as a substitute for systemic drug availability for medications that can only be given intravenously [12,59].

### 3.2. Nasal Delivery Challenges

Drugs administered via the nasal mucosa have several obstacles to overcome, including physiological, physicochemical, and mechanical ones, before they can be successfully absorbed. These obstacles include the following: the physicochemical challenges of the active ingredient; nasal geometry and the location of drug deposition; mucociliary clearance; drug transport across the respiratory epithelial membrane; diffusion through the mucus layer; potential enzymatic degradation; potential efflux drug transporters [69,70,71,72]. All of these challenges are generally associated with the morphology and physiology of the nose. Further, nasal formulations need to provide sufficient dosage in a small dose volume (100–150 µL per nostril) in order to be effective [73].

Furthermore, the nasal mucociliary clearance system protects the lower respiratory tract from dust and other particles. This system consists of goblet cell mucus and cilia [74]. The mucus moisturizes and shields the nasal epithelium physiologically. It moves posteriorly at a rate of from 1 to 20 mm/min and is continuously renewed every 10 to 20 min [14,75,76], with a pH range around 5.5–6.5; however, illness conditions may cause slight changes [77,78,79]. Substantially, mucociliary clearance removes particles from the nasal cavity to prevent them from entering the lower airways. Once particles and bacteria or viruses are trapped in the mucus, they will be transported backward by the ciliary beating to the pharynx for being swallowed or expectorated [69,80,81].

Moreover, the development of nasal drug administration should consider the relatively short residence time of drugs administered nasally and their potential degradation due to nasal secretions. This is critical because mucus can change the steady-state flux as well as the lag time [73].

In view of this background, it is important to note here that strategies for the successful delivery of nasally administered drugs are highly needed as there are various significant challenges through the nasal route pathway [82,83,84]. In this context, ensuring effective drug delivery through nasal route administration requires an appropriate formulation strategy. By considering the challenges and situations in the nasal passage, along with the intended therapeutic target, a suitable formulation strategy can be determined, one of which may come through the application of serum albumin nanoparticles as a nasal delivery carrier.

## 4. Serum Albumin in Nasal Drug Delivery

### 4.1. Application of Serum Albumin in Nasal Drug Formulation

Currently, there has been a lot of research that utilizes albumin-based nanoparticles as a carrier system to deliver active ingredients through the nasal route [85]. Albumin-based nanoparticles offer many beneficial aspects, either for the formulation itself or for the nasal applicability. In the aspect of formulation, albumin has a huge binding affinity (lipophilic or hydrophilic drug); therefore, various kinds of drugs can be easily soluble within albumin.

Moreover, if an albumin-based nanoparticle has a higher entrapment efficiency, then it will be efficient for embedding the drug. Also, albumin-based nanoparticles can increase the stability of the drug. Further, from the point of view of nasal applicability, as many challenges can be found in the nasal cavity, which definitely affects the formulation, the proper carrier system is of great importance. In order to avoid rapid elimination from the nasal passage, good mucoadhesive behavior of the formulation is needed. Albumin-based nanoparticles have shown great results in prolonging the in vitro residence time of drugs, which are evaluated in accordance with nasal conditions [9]. Also, albumin exhibits the ability to improve the mucoadhesive behavior of formulation while in contact with mucin in the nasal cavity. It has been previously studied that albumin has a special interaction with mucin; in this case, a specific albumin–mucin binding can occur, enhancing the adhesivity and avoiding rapid clearance due to the mucociliary process [86].

Furthermore, in an attempt to counteract the results of the ineffectiveness of the formulation due to some specific nasal conditions, certain polymers can be employed. In this regard, albumin can be formulated together with the polymer to achieve the desired properties for nasal applicability. As shown in Table 2, various studies have been conducted to utilize albumin-based nanoparticles for intranasal delivery.

The most common albumin types that have been utilized for nasal drug delivery are human serum albumin and bovine serum albumin. In this case, the specific method to prepare the albumin nanoparticles was also varied, including methods such as the coacervation method, desolvating method, and a simple mixture with water to investigate the release of the drug through the nasal condition. Further, based on this research, it can be seen that there are many types of API that could be entrapped in albumin-based nanoparticles. Also, stimuli-sensitive polymers can be utilized, like thermosensitive polymers and ionic-sensitive polymers, to utilize the specific condition of the nasal cavity, like the specific temperature or ionic conditions in which they can trigger the polymer to perform an in situ gelling system, which can then result in mucoadhesive properties in order to retain the formulation in the nasal cavity [85]. This formulation of drug-loaded albumin nanoparticles showed good release behavior, allowing it to retain a gel formulation in the nasal cavity.

Moreover, it has been previously determined that albumin has the ability to enhance the permeation of molecules through the nose and the brain barrier. If this is the case, it will provide a great opportunity to employ albumin for nose-to-brain route delivery, allowing for the treatment of various neurodegenerative diseases. For example, drug-loaded human serum albumin nanoparticles have been studied to evaluate their ability to permeate the brain barriers [8]. In this research, we were able to enhance the permeation of the drug through a specific BBB lipid fraction, indicating a possibility of delivering the drug across brain barriers.

Given the number of studies that have investigated the use of serum albumin as a nasal delivery carrier with their fascinating and promising findings, this may indicate that serum albumin could play a significant role in ensuring effective nasal drug delivery.

### 4.2. Role of Serum Albumin in Nasal Drug Delivery

Serum albumin has been extensively used as a carrier in nasal drug formulation due to its various advantages, especially the ability to enhance the drug retention time in the nasal cavity. In general, the mechanism by which serum albumin prolongs the retention time of nasal drug delivery involves several key factors, including its mucoadhesion ability, sustained release profile, minimized clearance, and targeted delivery [92]. In such a scenario, serum albumin possesses mucoadhesive properties, allowing it to adhere to the nasal mucosa. Interaction occurs between albumin and mucin, the primary glycoprotein component of mucus [93]. This adhesion prolongs the residence time of drug formulations within the nasal cavity. In this case, the utilization of serum albumin-based drug carriers, such as nanoparticles, can be formulated to incorporate the drug of interest.

The mucoadhesive nature of albumin ensures that the drug carrier remains in contact with the mucosa for an extended period [94]. Therefore, a sustained release can be achieved, and the drug can be gradually released over time. In particular, the sustained release profile is influenced by the interaction between albumin and mucin, as well as the specific formulation design. Also, the prolonged retention time prevents rapid clearance of the drug from the nasal cavity. This minimizes the risk of premature elimination and allows for gradual absorption. By remaining localized in the nasal mucosa, albumin-based carriers enhance drug delivery to the desired site of action. Targeting specific regions within the nasal cavity can improve therapeutic efficacy. Overall, serum albumin’s mucoadhesive properties, sustained release behavior, and ability to prevent rapid clearance contribute to prolonging the retention time of nasal drug formulations, ultimately optimizing nasal drug delivery.

In terms of the mucoadhesive behavior of serum albumin, the interaction with nasal mucin plays an important role. Generally, mucin consists mainly of water (approximately 95% *w*/*w*), mucins (approximately 0.2 to 5.0% *w*/*v*), globular proteins (approximately 0.5% *w*/*v*), salts (approximately 0.5 to 1.0% *w*/*w*), lipids (1–2% *w*/*w*), DNA, cells, and cellular debris. Mucin acts as a selective barrier to drugs and other molecules by covering epithelial cells in a thick, viscoelastic layer. A large number of physical entanglements stabilized by covalent and noncovalent interactions, such as hydrophobic, electrostatic, hydrogen bonding, or other specific binding interactions, are present in the mucus layer. These interactions enhance the viscoelasticity of the mucus and form a mesh network filter that reduces the rates at which molecules and particles diffuse and penetrate [95].

Mucins are very high molecular weight (10–40 MDa) glycoproteins that form polymeric gels and are secreted by submucosal glands and epithelial goblet cells. With a carbohydrate density of over 70%, mucin fibers are filamentous O-linked glycoproteins with “PTS” (proline, threonine, and serine) repeated domains that are highly glycosylated [96]. N-acetyl-galactosamine, glucosamine, fructose, galactose, sialic acid, and trace amounts of mannose and sulfate are the main components involved in glycosylation [97]. Since mucins are densely glycosylated, their arrangement resembles a brush. High calcium ion concentrations inside the secretory glands help in mucin condensation by protecting negatively charged sulfate and sialic acid groups [98].

Hydrophobic globular regions with a high cysteine content are scattered throughout the PTS domains, creating intra-disulfide bonds. Later polymerization results in the formation of long linear oligomers, which give the mucus layer its adhesive and swellable qualities [99]. Most mucin glycoproteins have high sialic acid and sulfate contents, which give them a strongly net-negative surface charge that increases stiffness by charge repulsion. Mucins transition from a random coil to an extended conformation at acidic pH values, forming a gel phase in mucus. It was suggested that these conformational alterations would promote hydrophobic interactions between mucin macromolecules at low pH levels, resulting in a sol–gel transition state [100,101].

Mucins are generally classified into two categories: secreted mucins and membrane-bound mucins. Secreted mucins are strongly associated with the viscoelastic characteristics of mucus, whereas membrane-bound mucins are linked to cellular adhesion, pathogen binding, and signal transduction processes. Humans have been found to have multiple mucin genes (MUCs). Regarding mucus in the airways, the two main polymeric mucins are MUC5A and MUC5B [96], which are secreted by mucous cells found in submucosal glands and goblet cells on the surface of the epithelium, respectively. These mucin genes are crucial for both the function of mucociliary clearance and the regulation of infections in the respiratory system [99].

**Figure 3:**
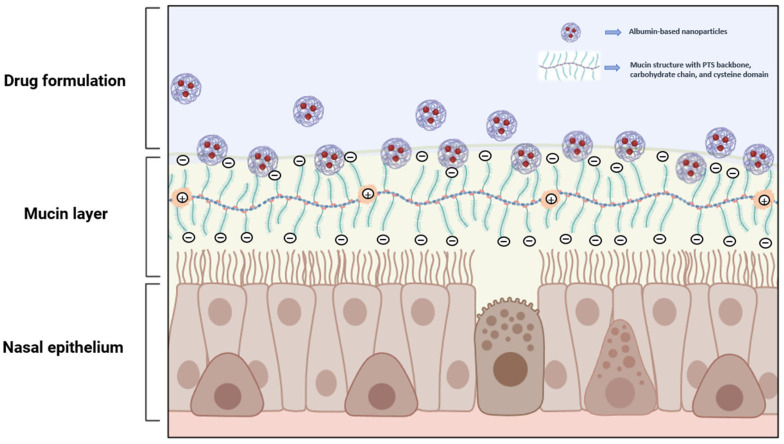
Schematic illustration of drug-loaded serum albumin nanoparticles while in contact with the negatively charged mucin layer on the nasal mucosa; albumin–mucin binding might be attributed to the electrostatic interaction.

It is significant to highlight here that the role of albumin in prolonging the retention time in the nasal cavity can be attributed to the formation of mucin–albumin complexes through the interaction between albumin and the nasal mucin layer, as shown in Figure 3. In this circumstance, mucin–albumin complexes can form because albumin binds to the nasal mucin actively through electrostatic interactions and primarily occurs at neutral pH conditions [86].

Notably, in a condition where the pH is above the isoelectric point of albumin (pH > pI) or at a neutral pH, the albumin molecules become negatively charged. In this case, specifically, the bonding interaction between mucin and albumin occurs due to the presence of a positively charged cysteine domain in the mucus structure [86,99,102]. Serum albumin with negatively charged molecules establishes associations and interactions toward the positively charged cysteine domain residues of mucin matrices, thus increasing the viscosity as well as the mucoadhesivity. This process is mainly driven by van der Walls, ionic, or electrostatic interactions, particularly when a highly negatively charged molecule is involved [93,102,103]. Further, the albumin–mucin complexes might facilitate a higher residence time in the nasal cavity, allowing a sustained release profile of the drug to be achieved. This release profile is very useful for nasal drug formulations intended for local therapy that require proper contact with the target site of action in order to release the active substance, for example, the treatment of acute bacterial rhinosinusitis [89]. Moreover, a sustained release profile is also beneficial for systemic therapy purposes since it can possibly enhance drug bioavailability, allowing the required drug concentration to be maintained for effective treatment [104].

As previously mentioned, it is known that albumin actively binds to mucin and that these interactions are noticeably stronger at a neutral pH. Therefore, in this instance, higher electrostatic interactions can significantly occur to promote the formation of mucin–albumin complexes [86]. On the other hand, when the pH is lower than the isoelectric point of albumin (pH < pI), albumin molecules acquire a positive charge, leading to the reduction of electrostatic forces with mucin. As a consequence, the binding to the mucin matrices might no longer exist. Thus, the viscoelastic network of mucin–albumin complexes is unable to be established, and the rheological properties are driven by weak hydrophobic interaction with mucin molecules [86]. The interaction between albumin and mucin particularly generates a mucoadhesion process, which can help in achieving complete absorption of the administered dose. Such interaction would be beneficial in nasal drug formulation for systemic delivery purposes or CNS-targeting, presenting rapid absorption while avoiding first-pass effect metabolism, leading to a higher drug bioavailability.

Moreover, regarding nose-to-brain delivery, albumin also plays an important role in carrying the drug of interest to cross the blood–brain barrier. It has been reported that serum albumin in a nanoparticulate form was able to reach the brain region to deliver R-flurbiprofen in an attempt to delay Alzheimer’s Disease progression [85]. The mechanism by which albumin can enter the brain has been previously studied. Albumin was rapidly absorbed from the nasal mucosa into the brain by means of transcytosis across the nasal epithelium and then transported paracellularly further into the brain [42,43,103]. On this basis, a dose-dependent mechanism, possibly involving fluid-phase transcytosis, allows intranasal albumin to reach the brain [43].

Moreover, the possible nanoparticle absorption through nasal mucosa involves either intracellular or extracellular mechanisms, in which drug molecules are mostly transported via the combination of those mechanisms. For the intracellular mechanism, the transport process begins with endocytosis through the neuron surface at the epithelium area, which is then followed by axonal transport and, subsequently, exocytosis in the CNS. Meanwhile, extracellular transport occurs through several mechanisms involving tight junctions (TJs) between the epithelial cells and the lamina propria. After that, the transport continues to the perineural space, blood vessels, and lymphatics [61].

Furthermore, the utilization of serum albumin in the form of nanoparticulate systems has gained considerable attention [3,6]. In this regard, the surface of albumin-based nanoparticles may contain reactive groups (thiol, amino, and carboxylic groups) that can be used for surface modification or drug–ligand conjugation. These nanoparticles are easily prepared and reproducible in defined sizes. The albumin nanoparticles may facilitate the electrostatic adsorption of positively or negatively charged molecules because of their high content of charged amino acids, such as lysine, arginine, aspartate, and glutamate. For example, a viable method for delivering drugs to tumor cells specifically is through the use of albumin nanoparticles, and the mechanism underlying their increased uptake in solid tumors is the binding of albumin to albumin-binding proteins, including membrane-associated 60 kDa glycoprotein (gp60 or albondin) and SPARC-mediated receptor endocytosis [25,48].

As a rule of thumb, the essential role of serum albumin is exerted through its specific interaction with nasal mucin, supporting the mucoadhesion process, coupled also with its penetrating ability to the brain area via the olfactory pathway, making serum albumin an attractive delivery carrier for nasal route administration. Therefore, serum albumin can be a versatile choice for preparing favorable nasal drug formulations, and, in this regard, the application can be performed through the fabrication of drug-loaded serum albumin-based nanoparticles.

## 5. Preparation Techniques of Serum Albumin-Based Nanoparticles

The preparation of albumin-based nanoparticles as drug carriers has been extensively studied. There are several techniques that can be employed, such as desolvation (coacervation), emulsification, thermal gelation, and, most recently, nano-spray drying, nanoparticle albumin-bound technology (nab-technology), and self-assembly.

### 5.1. Desolvation

Albumin nanoparticles are frequently prepared by a process called desolvation, which is a thermodynamically driven self-assembly process [105,106]. Typically, a turbid solution is achieved by gradually adding acetone or ethanol dropwise into an aqueous albumin solution while stirring continuously. Two liquid phases separate from the homogeneous solution phase; the solute (albumin) is present in one of the liquid phases, which is essentially pure solvent. The concept behind the formation of nanoparticles is that a conformational change in the protein structure reduces the solubility of the protein, causing the formation of a protein-rich phase known as the coacervates [107]. Sometimes, the morphologically formed albumin particles are not stabilized enough, and, as a result, coacervates are crosslinked (e.g., with glutaraldehyde) to harden them [108]. The initial protein concentration, pH, ionic strength, crosslinking agent concentration, agitation speed, and amount of desolvating agent are some of the preparation conditions that affect the properties of the resulting nanoparticles [106]. Nanoparticles prepared through this method can be stored for a period of time after the freeze-drying process, and the reconstituted nanoparticles can be then treated or modified with targeted ligands. Recently, this preparation technique has been widely employed in antitumor applications [109,110].

### 5.2. Emulsification

Albumin nanoparticles have been prepared using the emulsification technique extensively. A non-aqueous medium, like oil, is used to emulsify an aqueous protein solution. Both high-energy and low-energy emulsification techniques can be used to produce these emulsions [6]. Chemical or physical (thermal) crosslinking are used to stabilize albumin nanoparticles and protect their structural integrity. Following the emulsification process, albumin nanoparticles can be crosslinked chemically with agents such as glutaraldehyde, formaldehyde, or diacid chloride or thermally stabilized by heating (above 120 °C). Crosslinking agents can cause toxicity and unwanted reactivity with other chemical components, so it is imperative to remove them from the final nanoparticles. Drugs that are water-insoluble can be successfully encapsulated in nanoparticles made using the single emulsification method, but hydrophilic drugs cannot be effectively entrapped using this technique. As a result, the double emulsification method can be used to create more complex structures by adding the primary single emulsion to an aqueous solution of a second biopolymer. Proteins and peptides, among other highly water-soluble substances, can be effectively encapsulated by these nanoparticles [22]. The emulsification method has been widely applied in the production of polymeric nanoparticles. Albumin nanoparticles produced by this technique may be degraded due to ultrasonic action or mechanical shear force and may have a lower encapsulation efficiency than hydrophilic drugs.

### 5.3. Thermal Gelation Method

During the thermal gelation process, heat causes some polypeptide segments to unfold, which, in turn, causes protein–protein interactions such as hydrogen bonding, electrostatic interactions, hydrophobic interactions, and the disulfide sulfhydryl interchange reaction. The equilibrium between repulsive and attractive forces—which change depending on the molecular and physical characteristics of proteins—is necessary for the formation of a matrix. The nature and concentration of other solids, pH, ionic strength, protein concentration, and other variables can also have an impact on this formation and the texture that results [111]. The thermal gelation method produces albumin nanoparticles that are comparatively stable and have good mechanical properties. However, they are not appropriate for heat-sensitive substances. This technique has been widely used for preparing nanoscale hydrogel albumin-based nanoparticles [31].

### 5.4. Nano Spray Drying Method

The process of spray drying, which is widely employed in the pharmaceutical industry to create a dry powder from a liquid phase, involves spraying droplets made from a solution of the drug and protein. Four basic steps make up a typical spray drying process: atomizing feed into a spray, spray–air contact, spray drying, and separating the dried product from the drying air. It provides the benefit of continuous, scalable, single-step drying and particle formation. Nowadays, there is a new version of nano spray dryer that uses a vibrating mesh technology to generate fine droplets; meanwhile, conventional spray dryers form spray droplets using rotary atomizers (atomization by centrifugal energy), pressure nozzles (atomization by pressure energy), or two-fluid nozzles (atomization by kinetic energy). Thus, “nano” spray drying has become feasible with an electrostatic particle collector and vibrating mesh spray technology [6]. Nanoparticles prepared by nano spray drying typically have a high drug-loading efficiency. High siRNA-containing HSA-based powders have been made utilizing this technique [112].

### 5.5. Nanoparticle Albumin-Bound Technology (Nab^®^ Technology)

The FDA’s recent approval of nab-paclitaxel (Abraxane^®^, Abraxis BioScience LLC, Los Angeles, CA, USA) has generated significant interest in the field of anticancer drug delivery systems, primarily due to nab-technology. The emulsion evaporation crosslink method is the basis for the preparation of nab-paclitaxel. Drop by drop, the drug-containing oil phase is added to the albumin solution that has been pre-saturated with chloroform in the aqueous phase. When a mixture is subjected to low shear forces at low rotation speeds, a crude emulsion is formed. The crude emulsion is then homogenized using a high-pressure homogenizer to produce a final emulsion. Eliminating the solvent yields albumin nanosuspensions. There are currently a number of nab medications being developed, including ABI-008 (nab-docetaxel) and ABI-009 (nab-rapamycin), and it is believed that a comparable preparation process can be used for them [22,34]. Nab^®^ Technology has been utilized for fabricating hydrophobic drug-loaded albumin nanoparticles. The albumin nanoparticles loaded with drugs typically have a particle size between 100 and 200 nm.

### 5.6. Self-Assembly

Albumin can self-assemble into nanoparticles by becoming more hydrophobic through the addition of lipophilic medications, the breaking of disulfide bonds, or the reduction of primary amine groups on protein. The hydrophobic domains of albumin then unite with drug molecules to facilitate albumin’s self-assembly into nanoparticles. β-mercaptoethanol, cysteine, and dithiothreitol are examples of common reducing agents. Albumin nanoparticles produced via the self-assembly method have active targeting characteristics because they can better retain the functions of the protein. Previous research has utilized the self-assembly of bovine serum albumin to prepare nanoparticles loaded with doxorubicin [53].

## 6. Future Prospects

The application of serum albumin for the successful delivery of nasal drug administration through the utilization of albumin-based nanoparticles offers great potential for the future development of nasal drug administration. In this case, targeted drug delivery can be supported by functionalizing albumin nanoparticles with targeting ligands, and specific tissues or cells can be selectively addressed. Hence, this precision enhances therapeutic efficacy while minimizing off-target effects. Moreover, as albumin-based nanoparticles offer a potential route for delivering drugs to the central nervous system (CNS), then it is of great importance to explore their use in treating neurodegenerative diseases and brain tumors [113].

In line with this, a combination therapy might also take place. Albumin nanoparticles can carry multiple drugs simultaneously. Combination therapies for cancer, infections, or other diseases can benefit from this approach. Therefore, the application of smart polymers and responsive systems can be great advantages. The combination of albumin-based nanoparticles with stimuli-responsive properties such as temperature, pH, and enzymes can also be used. These “smart” systems allow for controlled drug release and adaptability to nasal conditions. In the future, personalized medicine might not be seen as something impossible, and tailoring albumin nanoparticles to individual patient needs may become a reality. Customized drug delivery systems could optimize therapy outcomes.

## 7. Conclusions

Serum albumin possesses many beneficial roles for nasal drug delivery. As nasal route delivery generates certain challenges for drug formulation, the application of serum albumin can be useful, specifically for enhancing mucoadhesive properties by maintaining the ability of the drug to bind with mucin in the nasal cavity, therefore hindering rapid clearance due to nasal mucociliary clearance and gaining appropriate drug bioavailability in the target site. Moreover, for brain targeting purposes, albumin also has great potency in helping the drug to pass the condition in olfactory nerves, allowing great permeability to the brain region. Considering the great role and potential of serum albumin for nasal drug delivery, and in order to guarantee its successful utilization as well as to gain its advantageous properties for nasal drug administration, a proper understanding and knowledge regarding serum albumin are of great importance.

## Figures and Tables

**Table 2:** Various studies employing albumin-based nanoparticles for intranasal delivery.

Sr. No	Albumin Type	Preparation Method	Research Purposes	API	Results	Ref.
1	Bovine serum albumin	Coacervation method	Plasma profile evaluation —bioavailability	Silybin (SLB)	Intranasal administration of BSA-Nanoparticles (NPs)/SLB in rats improved SLB bioavailability by fourfold compared to free SLB	[87]
2	Bovine serum albumin	Desolvation method	Local nasal therapy—ABR	Amoxicillin trihydrate (AMT)	AMT-loaded BSA NPs with in situ thermosensitive polymer inhibited the growth of ABR pathogens	[88]
3	Bovine serum albumin	Desolvation method	Local nasal therapy—ABR	Amoxicillin trihydrate (AMT)	AMT-loaded BSA NPs with in situ ionic-sensitive polymer inhibited the growth of ABR pathogens	[89]
4	Bovine serum albumin	Desolvation method	Nose-to-brain—Alzheimer disease	Tacrine hydrochloride	Albumin-based NPs with hydrophilic derivatives of betacyclodextrin showed an interesting drug permeation profile	[90]
5	Human serum albumin	Desolvation method	Nose-to-brain	Sulforhodamine B sodium salt	The formulation was able to influence the tight junction, allowing permeation of the molecules	[91]
6	Human serum albumin	Coacervation method	Nose-to-brain—Neuroinflammation	Meloxicam (MEL)	Enhanced permeation of MEL was detected through specific BBB-lipid fraction	[7]
7	Human serum albumin	Coacervation method	Nose-to-brain—Neuroinflammation	Meloxicam (MEL)	Higher cerebral concentration of MEL was observed	[8]
8	Human serum albumin (Albutein 20%)	Simple mixture with water	Nose-to-brain—Alzheimer disease	R-Flurbiprofen (R-FP)	In vivo brain concentration albumin-based nanoparticles of R-FP was higher compared to intranasal and oral R-FP solution	[85]
